# Editorial: Insights in pediatric pulmonology 2021

**DOI:** 10.3389/fped.2022.1093793

**Published:** 2022-11-23

**Authors:** Federica Porcaro, Renato Cutrera

**Affiliations:** Pediatric Pulmonology & Respiratory Intermediate Care Unit, Sleep, and Long Term Ventilation Unit, Academic Department of Pediatrics (DPUO), Bambino Gesù Children’s Hospital, IRCCS, Rome, Italy

**Keywords:** BPD (bronchopulmonary dysplasia), bronchiolitis, infections, CF (Cystic fibrosis), ACT (airway clearance techniques), lung function test, sleep disorders

**Editorial on the Research Topic** Insights in pediatric pulmonology 2021

Respiratory diseases are the commonest cause of morbidity in paediatric age in developed countries (1). Paediatricians must increasingly gain the knowledge, skills and experience they will need to treat a broad range of conditions that vary from acute and chronic respiratory disorders in children and adolescents. Because there is the urgent need to keep up with the research, we aimed to highlight the latest news in Pediatric Pulmonology.

We know that the advances in neonatal intensive care have led to improved survival in preterm infants (2). The effect of this result is the increase of infants affected by chronic conditions that affect primary the lung, but also undermine the balance of the whole organism. Broncho-pulmonary dysplasia (BDP) is an example of a neonatal chronic lung disease responsible of significant morbidity and mortality in preterm newborns (3, 4). The most accepted definition of BPD is based on the requirement of oxygen supplementation either at 28 days postnatal age or 36 weeks postmenstrual age (5). The aetiology of BPD is multifactorial and involves disruption of lung development and injury due to antenatal and/or postnatal factors (6). Some studies support the hypothesis that the development of BPD begins before birth after the exposition to proinflammatory cytokines during the intrauterine life. The exposure to inflammatory mediators may disrupt lung development through several mechanism (increase of vascular permeability, protein leakage, and mobilization of neutrophils into the interstitial and alveolar compartments). The increase of pro-inflammatory cytokines and the decrease of the counterregulatory ones may lead to unregulated and persistent inflammation (5). Wang et al. carried out a retrospective study to establish the association between levels of inflammatory cytokines in cord blood and bronchopulmonary dysplasia (BPD) in preterm infants. The results of the analysis carried out on 147 premature infants with gestational age ≤32 weeks showed that IL-6 cord blood levels at birth are significantly higher in the BPD group than in the non-BPD group. Therefore, Authors concluded that increase levels of IL-6, as well as increase maternal white blood cell (WBC) count on admission and lower birth weight, increase the risk of BPD progression.

Infectious respiratory illnesses are more frequently reported by parents in the first years of life (7). Acute viral bronchiolitis is one of the leading causes of hospitalization in the first 12–24 months of life (8). Airway cleansing, oxygen support and rehydration represent the main strategy suggested by international guidelines to manage viral bronchiolitis (9). As recent studies describe different phenotype corresponding to specific endotypes, trial with bronchodilator has been proposed in children with phenotype of bronchiolitis more strongly associated with asthma features. As reported by Bottau et al., the last one is likely in those infants with the first viral induced wheezing and aged over 6 months.

Beyond viral respiratory infections, bacterial acquired pneumonia sustained by Mycoplasma pneumoniae (MP) is often reported in school aged children (10). MP infection can cause serious consequences. Qiu et al. carried out a retrospective study to find the risk factor for developing severe MP pneumonia (SMPP). Based on the results of this research study, the percentage of neutrophils, the platelets count, the levels of C-reactive protein (CRP), lactate dehydrogenase (LDH) and D-dimer are risk factors for SMPP. Among the above-mentioned factors, D-dimer is the best predictor for complications such as pleural effusion, myocardial and liver damage.

According to the World Health Organization (WHO) 1 million children per year have TB disease and many more have a latent form of infection (11). Though treatment is resolving, some children may develop pleural tuberculosis and sometimes tuberculous empyema (TE). As the cause of TE in TB infected patients is unknown, Wu and colleagues designed a retrospective study to assess the factors associated with the presence of TE in children. The Authors concluded that surgical treatment, lung cavitation, high pleural LDH level, and low temperature are risk factors of development of TE in children with pleural TB.

Long lasting respiratory symptoms, like chronic cough, can be troublesome for paediatricians, also in a specialized setting (12). Despite advances in diagnostic imaging e laboratory tests in the field of respiratory medicine, history taking remains a primary step in diagnosing children with chronic cough. Kantar et al. stressed this concept in their kindly and accurate review, confirming the motto “history is half the diagnosis”.

Among respiratory conditions to consider in the differential diagnosis of chronic cough, cystic fibrosis (CF) is one of the most feared diseases to exclude especially in children with other associated symptoms like frequent expectoration, bronchiectasis on chest CT and growth failure (13).

Like reported by Galodé et al., wheezing is not infrequent in CF affected children aged under 6 years, and bronchodilator response (BDR) is most represented in the group of patients aged 6–8 years. Atopy and Pseudomonas aeruginosa colonization in CF pre-schoolers are not associated with risk of wheezing or BDR. Nevertheless, CF affected wheezers at the age of 6 years have a worse lung function when compared with CF peers without wheezing.

Airways clearance techniques (ACT) are certainly a key point treatment in CF affected children. Its usefulness in the long-term period is undisputed (14, 15). Vandervoort and colleagues questioned if a single ACT session using positive expiratory pressure (PEP) mask has a short-term positive effect on forced expiratory volume in 1 s (FEV_1_) and lung clearance index (LCI) in children affected by CF and primary ciliary dyskinesia (PCD). Despite the small sample, the Authors concluded that single PEP mask session has no significant short-term effect on FEV_1_ and LCI in the analysed groups.

Anyway, other airways clearance techniques can improve lung function and physical resistance, finding application in certain conditions including obesity (16). Indeed, Kaeotawee et al. compared the effect of threshold inspiratory muscle training (IMT) on functional fitness and respiratory muscle strength (RMS) with incentive spirometry in 60 obese children and adolescents aged 8–15 years. The Authors concluded that 8 weeks of IMT training distance improve six-minutes walking test (6-MWT) and maximal inspiratory pressure (MIP) in obese paediatric patients.

It is well known that pulmonary function tests are important tools to diagnose and monitor chronic lung diseases (17). Although Radics et al. claimed their utilization in newborns is still far from being defined, Chaya et al. verified that spirometry remains the commonest test used in school age also in low-middle income countries. When associated with salbutamol test, spirometry allows the detection of bronchial reversibility that is typical of asthmatic patients (18). Asthma is the most common chronic disease in childhood, affecting an estimated 7 million children (19). Although most patients have good control of asthma symptoms, some children attend emergency department (ED) for acute asthma attack (20). Usually, the poor adherence to prescribed therapies justifies the need to access the hospital for acute attack. As reported by Kennedy and colleagues, the periodical revision of correct inhaler technique and the drafting of a personalised care plan have proven to be effective to reduce the ED and the systemic steroids utilization for asthma exacerbation.

However, it is reported that asthma can remain severe in a subtype of children despite the optimization of treatment and good adherence. For this group of patients, understanding of underlying molecular mechanisms is useful to develop a target therapy (21, 22). To date, as suggested by Ghirardo et al., the same molecular approach used for asthma treatment may be considered to treat other paediatric respiratory diseases for which the molecular basis is known.

Finally, up to 50% of children will experience a sleep problem (23). Early identification of sleep disorders may prevent negative effects of sleep disturbance, such as irritability, behavioral problems, daytime sleepiness and learning difficulties (24). Most studies on sleep disorders in paediatric age are related to children living in low altitude. Ucrós and colleagues proposed a recent review on sleep disorders in children living in high altitude. They concluded that central apnea index decreases with age, while obstructive apnea/hypopnea index has a biphasic course, and periodic breathing in the first months of life is more marked with increasing altitude.

Among sleep disorders, obstructive sleep apnea (OSA) occurs in 1%–5% of children (25). Continuous positive airways pressure (CPAP) is the standard treatment (26). However, devices that automatically adjust the pressure during sleep (automatic PAP) are available (27). Tovichien et al. investigated the adherence and tolerance to CPAP and automatic PAP in a subgroup of paediatric patients affected by OSA. They found no statistically significant differences in the adherence and tolerance of the children using these devices.

## Conclusions

This Topic offers a comprehensive update about several common respiratory conditions. We have a very special reason to be proud of this Issue as it represents a useful tool for clinicians in their clinical practice, also in not specialized setting.
